# The lived experience of anxiety and the many facets of pain: A qualitative, arts-based approach

**DOI:** 10.1080/24740527.2020.1720501

**Published:** 2020-09-24

**Authors:** Roberta Lynn Woodgate, Pauline Tennent, Sarah Barriage, Nicole Legras

**Affiliations:** a College of Nursing, Rady Faculty of Health Sciences, University of Manitoba, Winnipeg, Manitoba, Canada; b School of Information Science, College of Communication & Information, University of Kentucky, Lexington, Kentucky, USA

**Keywords:** youth, pain, anxiety disorders, qualitative research, open-ended interviews, photovoice, ecomaps

## Abstract

**Background**: Findings reported in this article emerged from the study titled “Youth’s Voices: Their Lives and Experiences of Living with an Anxiety Disorder.” Though the initial focus of this study was not on the pain experiences of youth living with an anxiety disorder, it became apparent from the very first interviews that pain and suffering was key in the youth lived experience, permeating their everyday lives and impeding their participation and functioning in the world.

**Aims**: The aim of this article is to highlight the ways in which pain is a central experience for young people living with an anxiety disorder.

**Methods**: The study was approached from the qualitative research design of hermeneutic phenomenology. Fifty-eight young people who were living with anxiety disorders and their parents participated in the study. Youth took part in multiple qualitative open-ended interviews and the participatory arts-based method of photovoice. Themes were developed using van Manen’s method of data analysis.

**Results**: The overall theme emerged as “anxiety is very much about pain.” The four subthemes are (1) embodied experience of anxiety: physical pain; (2) a prominent symptom of anxiety: mental–emotional pain; (3) difficult interpersonal relationships: social pain; and (4) articulating their pain.

**Conclusions**: Use of qualitative, arts-based methodologies provided the opportunity and space for youth with anxiety to articulate their multifaceted experience with pain in their own words.

This work reinforces the need for use of qualitative approaches to understanding pain experiences in young people.

## RÉSUMÉ

**Contexte**: Les conclusions présentées dans ce document sont issues de l'étude intitulée « La voix des jeunes : Leurs vies et leurs expériences de la vie avec un trouble anxieux ». Bien que l'objectif initial de cette étude n’était pas de s'intéresser aux expériences de douleur des jeunes vivant avec un trouble anxieux, il est apparu dès les premiers entretiens que la douleur et la souffrance étaient des éléments clés de l'expérience vécue par les jeunes, qui imprégnaient leur vie quotidienne et entravaient leur participation et leur fonctionnement dans le monde.

**Objectifs**: Le présent document vise à mettre en lumière les façons dont la douleur constitue une expérience de premier plan pour les jeunes vivant avec un trouble anxieux.

**Méthodes**: L'étude a été abordée à partir de la conception de la recherche qualitative de la phénoménologie herméneutique. Cinquante-huit jeunes vivant avec des troubles d'anxiété et leurs parents ont participé à l'étude. Les jeunes ont pris part à de multiples entretiens qualitatifs ouverts et à la méthode participative Photovoice, fondée sur les arts. Les thèmes ont été déterminés en utilisant le processus d'analyse des données inspiré de van Manen.

**Résultats**: Le thème général qui s’est dégagé est le suivant : « L'anxiété est essentiellement liée à la douleur ». Les quatre sous-thèmes sont les suivants : (1) L'expérience de l'angoisse incarnée : La douleur physique ; (2) Un symptôme important de l'anxiété : la douleur mentale et émotionnelle ; (3) Les relations interpersonnelles difficiles : la douleur sociale ; et (4) l’articulation de leur douleur.

**Conclusions**: L'utilisation de méthodologies qualitatives fondées sur les arts a permis aux jeunes anxieux d'exprimer leur expérience multidimensionnelle de la douleur dans leurs propres mots.

Ce travail renforce la nécessité d'utiliser des approches qualitatives pour comprendre les expériences de la douleur chez les jeunes.

## Introduction

Pain is an inherently private aversive sensory and emotional experience to which only the individual has direct access.^1,2^ For young people living with pain, consideration of *how* their pain is understood is critical because it is a strong determinant in whether a child’s pain is appropriately treated.^3,4^ Young people living with pain experience a tension between describing pain in enough detail to be taken seriously and avoiding too much detail as to risk having the authenticity questioned.^5^ One in ten youth living with pain has experienced pain dismissal; over half of these instances involved a parent (52%) and at least a quarter involved a physician (30%).^3^ Youth and especially their parents have revealed that they feel a need to downplay nonphysical pain aspects in order to substantiate the pain.^6–9^ The lack of objective signs and inability to standardize pain have long challenged its epistemic value in health care and general society.^1^

Stories of pain in popular culture usually involve acute, visible trauma; curative treatment; and recovery.^1^ Over time, repetitive portrayals of such pain stories become mainstream belief and shape expectations.^1^ Lay people and health care professionals alike inherit a tendency to use binary oppositions to frame pain, deeming a young person as “well” or “ill” based on the visibility and acuity of their pain and their condition.^1^ Young people living with pain commonly hide their pain because they feel misunderstood, unsupported, and stigmatized by peers, parents, and health care professionals.^3,10,11^ The residual influences of biomedical and neurocentric perspectives are apparent in health care professionals’ tendencies to dichotomize pain as mostly physical or psychological in nature and their favoring of pain that is physically based.^12–16^

The pain experience of a young person living with mental illness is rarely acknowledged due the invisible nature of his or her disorder and the health care professional’s preference for physical pain. This is despite anxiety being the most commonly diagnosed mental health condition in Canada, affecting approximately 10% of children and adults across the country.^17^ For young people, anxiety can negatively shape all spheres of life, including school, their home lives, and peer relationships.^18,19^ Over 95% of young people living with an anxiety disorder also report experiencing symptoms such as headaches or stomachaches,^20^ yet reference to pain for people living with anxiety is rare in the literature. The experience of living with anxiety exists in a silo from any experience of pain, viewed as separate clinical paths.^21^ In part, this lack of understanding in the interaction between anxiety and pain may stem from not approaching anxiety from a biopsychosocial approach, in which pain is influenced by physical, psychological, environmental, and social dimensions.^12^ When assessed from a biopsychosocial standpoint, pain interacts reciprocally with anxiety in a manner that complicates the course of both conditions. Pain is a predictor of anxiety that persists longitudinally,^22,23^ whereas anxiety is a predictor of persistently elevated pain intensity and pain that increases over time.^24^ In the literature, there is evidence suggesting that social pain,^21,25^ emotional pain,^26^ and mental pain^27^ share neural pathways with physical pain, thereby potentially becoming modulating factors for one another.

Contributing to this lack of understanding into these complex phenomena is the emphasis on quantitative research. To date, research with young people living with visible, physical forms of pain and research on mental illness have primarily been approached by quantitative methods that are not well equipped to capture complex phenomena.^28^ Qualitative methods are valuable for capturing the unique reality of a young person living with an illness.^29^ The capacity of qualitative research to bring forth a new witnessing of pain in youth is a first step in provoking us to rethink what we know about pain and how we come to know it. Youth living with illness express a desire for others to understand “what am I feeling inside”^10(p1022)^ and describe it in enough detail to be taken seriously, yet this experience is often beyond words.^1^ Qualitative, arts-based methods have been shown to be helpful in facilitating self-expression of experiences, feelings, and perceptions not easily articulated by words.^1,30^ Arts-based methods like photovoice that capture personally significant experiences^31–33^ and/or ecomaps that display the nature of networks^34,35^ provide insight into the lived experience, contributing to the qualitative design of hermeneutic phenomenology. This methodology is a powerful strategy for accessing youths’ conscious and unconscious feelings about difficult life situations,^31,36–38^ such as misdiagnosis, stigma, and/or under-treatment.^1^ Literature reviews have proven qualitative, arts-based methods to be useful and valid for translation purposes in health care research.^30,39^ Capturing experiences adequately and accurately is an important step in validating health and illness models, including chronic pain models in children and youth.^40,41^ Woodgate and colleagues have provided support for the effectiveness of qualitative, arts-based methods in understanding health and illness experiences in young people by using drawing^36,42^ and photovoice.^36,37,43–49^

The aim of this article is to highlight the ways in which pain is a central experience for young people living with an anxiety disorder. This work demonstrates how qualitative, arts-based methodologies provided the opportunity and space for youth with anxiety to articulate their multifaceted experience with pain in their own words.

## Methods

### Research design

The qualitative research design of hermeneutic phenomenology was used. Hermeneutic phenomenology is both a research philosophy and a methodology. The philosophy, as informed by Merleau-Ponty,^50^ and the method, as informed by van Manen,^51^ guided the “Youth’s Voices: Their Lives and Experiences of Living with an Anxiety Disorder” research study. Hermeneutic phenomenology posits that our experience in the world is full of meaning, thus providing an opportunity to understand how living with anxiety shaped youth’s experiences from their frames of reference and experiences of reality. Ethical protocols included a review from the university’s research ethics board, written prior and informed consent for participants over the age of 18 and assent for participants who were minors, and an ongoing process of verbal consent.

### Participants

The selection of participants followed the maximum variation technique of purposive sampling.^52^ The power of purposive sampling lies in selecting information-rich cases for in-depth study.^53,54^ Youth participants were recruited from both clinical and community settings. In a clinical setting, youth were recruited from the waiting list of hospital-based programs that specialize in the treatment of anxiety disorders. In the community setting, youth were recruited from youth centers, teen clinics, and schools and via social media. In order to arrive at an accurate, comprehensive description and interpretation of the lived experience of anxiety, it was necessary to recruit 58 young people who were living with one or more anxiety disorders and their parents/guardians. Though 58 participants is large for phenomenology, it was important to gain a comprehensive understanding given that this was one of the first research projects in Canada that sought to detail the lived experience of young people with anxiety, thus justifying a larger sample size.^55–57^ Moreover, there was great interest in the project among young people whose anxiety typically could prevent them from participating in daily life. We also had appropriate resources available with which to handle the large sample size and permit deep and robust engagement with the data. The anxiety disorder diagnoses included separation anxiety, specific phobia, panic disorder, agoraphobia, social anxiety disorder, and generalized anxiety disorder, ranging from moderate to severe. The length of time with which participants had been living with their anxiety diagnoses varied; however, as a common theme, almost all participants stated that they felt that they had lived with traits of anxiety previous to any diagnosis. Participants were located in Winnipeg, Canada, and were between the ages of 10 and 22 years old at the time of the study, with a mean age of 14.5 years. Overwhelmingly, participants in the study identified as female (75%) and white (69%).

### Procedures

All participants took part in qualitative open-ended, face-to-face interviews. In-depth open-ended interviews afforded the opportunity to gather rich and detailed descriptions of the meaning of and experience of living with anxiety and provided youth the possibility to speak about topics they considered important and not anticipated by the researchers (in this case, their experiences with pain).^54,58^ Youth participated in more than one interview, an essential feature of a hermeneutic phenomenology in that repeated interviews allow for follow-up questions that add to issues identified in the initial interview.

An interview guide was used for the first interview. The interview guide had open-ended questions developed to get at what it is like to be a youth living with anxiety. The first interview was supplemented by asking youth to draw ecomaps. Ecomaps depict graphic portrayals of social relationships or networks of individuals or families, as well as places and activities.^34^ Prior to asking questions about how living with an anxiety disorder shapes daily life, youth were asked to draw circles that represent people, activities, and places that are and have been a part of their lives. Youth were also asked to draw lines between the circles that are meant to indicate the degree of connection that they have with each person, activity, or place represented on the map. Different types of lines represented different types of connections. The ecomaps were used throughout the first interview to promote discussion. Additional interview strategies included the use of silence, calls for examples, and probes to extend something the participant has said.^59^ When probing youth, great care was taken to ensure that the questions remained close to youth’s experiences.

The second interview was supplemented by the participatory arts-based method of photovoice, which involved youth taking photographic images to document and reflect on their experiences of living with anxiety. Photovoice is viewed as a useful strategy for obtaining experiential descriptions from individuals in phenomenology, providing a sense of being there and seeing the setting and people firsthand.^33,51,53^ It is an especially effective strategy for youth living with an anxiety disorder, because it is an unobtrusive way of entering the worlds of youth and can give meaning to thoughts and feelings that are often difficult to express with language.^59–62^ Woodgate has found that giving youth the opportunity to express and record their experiences through a camera is an excellent medium to facilitate sharing of their lived experiences, especially for those who may have difficulty elaborating on their thoughts in the moment.^31^ Photovoice gives youth increased control over the research process in that youth get to select what will be photographed and which photographs will be discussed in the interview setting. The photographs and ecomaps serve as graphical representations of the text-based findings^63,64^ and therefore help to contribute to a more inclusive understanding of the experiences of youth.

At the end of the first interview, youth were given digital cameras and asked to take pictures over a 3- to 4-week period of objects, people (if they obtained permission from them), places, or events that depicted their thoughts and feelings related to living with anxiety. Youth were then individually interviewed by means of the SHOWeD method, an approach that encourages discussion with participants on the meaning of the photos.^65^ The SHOWeD method involved guiding youth through their photos by asking them to identify what they see, to describe what they think is happening in each photo, and to explain how each photo relates to their lives and what each photo means to them in terms of living with anxiety. In addition, youth were asked follow-up questions based on their initial interview and to comment on any changes since the first interview.

All interviews were conducted at a private location that was most convenient and comfortable to participants (i.e., mainly in their homes). The interviews involved the youth participant and a research assistant trained by the first author. Interviews ranged in length from 30 min to 3 h. After each interview, field notes were recorded detailing the context of the interview. All interviews were digitally recorded and transcribed verbatim to preserve their authenticity.

### Analysis

As is common in qualitative research, data collection occurred concurrently with data analysis. This iterative approach, coupled with multiple data collection activities and research interviews, provided an opportunity for research participants to have ongoing input into theme development. All data emerging from interviews, photographs, ecomaps, and field notes were included in analysis, which was informed by van Manen’s method of data analysis.^51^ We first became fully immersed in the data by reading and rereading the interviews and field notes with attention to identifying significant statements, sentences, or sentence clusters that stood out as thematic of the lived experience of youth with anxiety disorders. Units of meaning were delineated and formed into thematic statements and themes were extracted. The graphical data (i.e., ecomaps and photographs) helped to inform the themes emerging from the youth interview data and field notes by formally linking the graphical data to the corresponding interview data. In short, the ecomaps and photographs served as graphical representations of the text-based findings and contributed to a greater understanding of the experiences of youth.^66,67^

## Results

The overall theme emerged as “anxiety is very much about pain,” which was supported by four subthemes: (1) embodied experience of anxiety: physical pain; (2) a prominent symptom of anxiety: mental–emotional pain; (3) difficult interpersonal relationships: social pain; and (4) articulating their pain.

### Anxiety is very much about pain

The initial goal of this research study was to capture the lived experiences of young people living with an anxiety disorder, with an emphasis on how they cope with and manage their anxiety. In this regard, the research study aimed to fill a gap in the research literature on anxiety disorders, which has been primarily quantitative in nature. A qualitative approach allowed for us to hear from the perspectives of young people, allowing them to share their experiences in their words and images. There were no questions in the interview guide specific to pain, yet it became apparent from the very first interviews that pain and suffering was key in the youth lived experience, permeating their everyday lives and impeding their participation and functioning in the world. One youth participant, who was 19 years old at the time of the interview, tried to sum up her experiences living with anxiety and depression and the invisible nature of those disorders:

[Anxiety and depression is …] like watching someone with their leg broken or something, crying out of pain and not, and not being able to do anything, you just don’t know what to do. But at least with your leg you can go to the hospital, you can figure out how to deal with it. But with being depressed or anxious, it’s like watching someone or, like, a baby cry constantly or watching a baby, like, ripped out of his mother’s arms, like, that’s how much anxiety and depression hurts. It’s just so painful and you feel like it hurts your heart, like it fully, like, aches and it just, it just hurts.

In describing their lives with anxiety, youth spoke about the complexity of pain and its impacts on both the mind and the body, breaking down the traditional dichotomy between the two. Youth also reflected on the various types of pain—physical, social, and mental–emotional—and the ways in which they interact with each other to cause suffering and damage to one’s self, often in a cyclical nature. A 17-year-old participant challenged the limitation of pain to that which is only physical, stating:

’Cause if you really think about it, it’s your mind that controls, like, your nerves and, like, your feelings and stuff like that. So pain is a mental thing. … It’s not only a physical thing because if it weren’t for your brain you wouldn’t feel it.

The divisions between the various types of pain were in some ways artificial, yet youth also relied on these divisions to describe their experiences. One 11-year-old participant shared,

Anxiety hurts; well, for me it hurt physically. … And I don’t know, I guess it hurts emotionally.

Another participant, 14 years old at the time of the interview, shared:

I feel pain, like, I don’t know it just, I feel sore about it, I feel weak, like I can’t do anything, it brings me down. … Like I have no control over it, like, once I’m, if I wake up sad it’s like I have no control over that and I’m just like that all day. But I try to get through.

**Figure 1.**
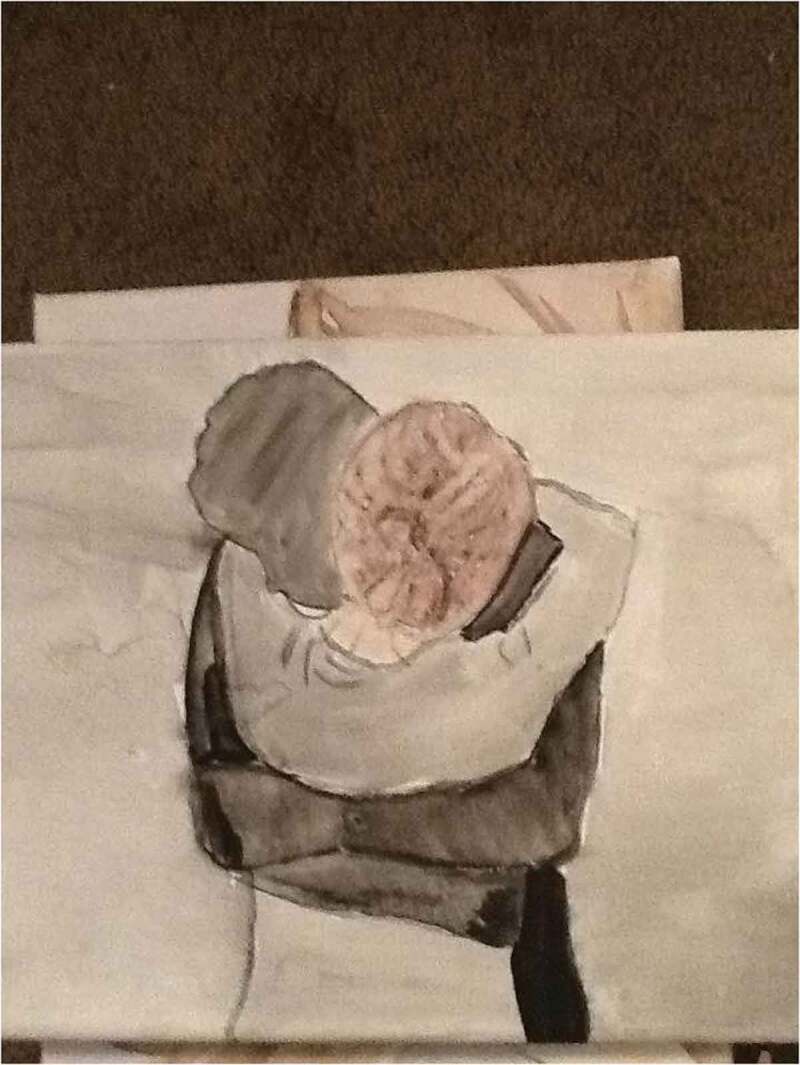
Anxiety—Your best friend that hurts you.

The various types of pain associated with anxiety impacted the sense of self of many of the participants in the study. Many of the young people had a negative image of themselves and how they viewed themselves in the world, which is in itself a form of emotional pain. Youth spoke of feeling disappointed in themselves, despising themselves, and experiencing emotional pain. They also described the ways in which their identity became tied to their mental illness and the challenges that come when that identity marker is linked to pain, as reinforced by this 14-year-old participant’s submitted photograph of her artwork (Figure 1):

I was sketching on a canvas and did this person. They are hugging their anxiety and I feel like some people make anxiety their friend. And they can be best friends, but your best friend is hurting you. Um, if a person is not good to you and not treating you right, you kind of have to let them go. … Just like sometimes it comes back and it will kind of suffocate you, but you have to still remember like it’s not your friend. … I painted it black. Because, like, that’s how I picture it [anxiety], it’s kind of like a dark cloud.

Young people shared that any attempts to deal with only one type of pain often served to accentuate all other types of pain, including that which was most visible and apparent: physical pain.

#### Embodied experience of anxiety: Physical pain

Anxiety manifested itself in the lived body of youth in a number of ways., Youth experienced a number of physical symptoms, such as chronic headaches, stomachaches, chest pain, and muscle aches, resulting from their anxiety. These physical symptoms, when combined with mental and emotional discomfort and distress, often lead to youth feeling overwhelmed by the pain, fearful of the pain, and fearful in anticipation of future pain and anxiety, alongside feelings of panic that they were losing control of their mind and their body. One 19-year-old, reflecting on the images of mental illness depicted online, shared:

You see these images of depression, with people, like, holding their head, and its not accurate. It’s like you’re in physical pain, but mental illness is on, like, a mental level plus a physical level. ’Cause, like, it wears out your body and, like, makes you feel heavy.

**Figure 2.**
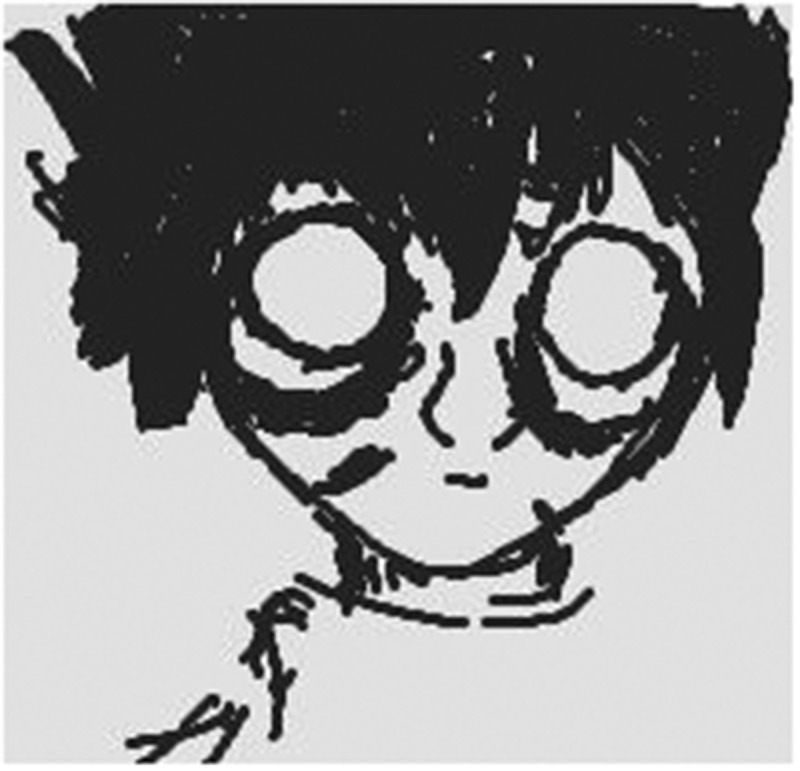
“Rough around the edges.”

Using photovoice, a 13-year-old male shared the following image and description of the ways in which anxiety impacted his physical self as he talked about his emotional pain (Figure 2):

Well, it kind of looks how I feel sometimes, sort of angry, sort of, kind of, almost beat, beaten up, or by “beaten up” I don’t mean literally so, but, like, kind of rough around the edges, you could say. … ’Cause that’s how I feel when I come home from a day after school.

**Figure 3.**
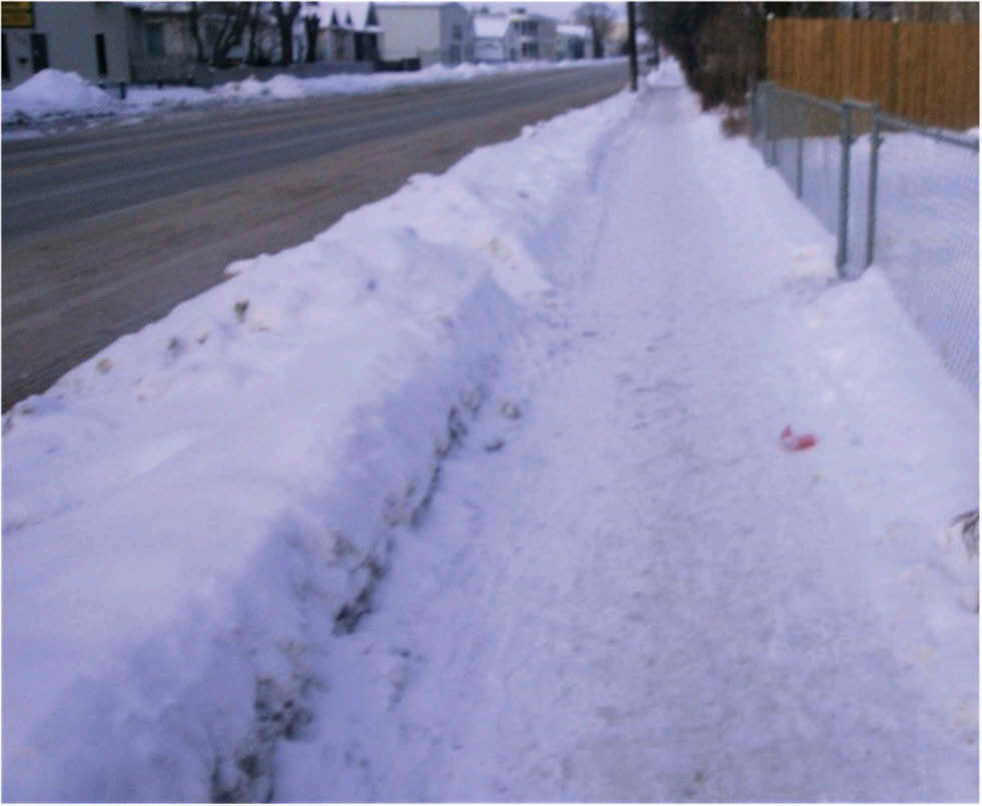
It hurt bad.

Another participant, 16 years old, returned to the site of where of his first panic attack to photograph the location for the purpose of this project. He recalled the pain and accompanying fear that resulted from the panic attack (Figure 3):

This is, this is one of the places I first had my anxiety attack. … Like when I was 14. I was just walking down the street and then these guys walked past me and they’re like … “What’s up?” And I started panicking. … And, um, I, I knelt down and I was breathing hard and my heart was beating fast. … And it felt and it hurt bad, like, in my chest.

#### A prominent symptom of anxiety: Mental–emotional pain

For many participants, living with an anxiety disorder was characterized as feeling a constant state of worry, stress, overthinking, and feelings of being out of control. As a result, participants felt that mental–emotional pain was prominent in their lives, even more so than the physical pain. For youth participants, this mental–emotional pain was not equated with the actual disorder of anxiety or depression but rather as a symptom of those disorders. One 13-year-old participant, in trying to explain why completing their homework took significantly longer than it should have, described the interaction of anxiety and mental pain as follows:

**Participant:** Well, my schoolwork now takes 4 hours on a weekday. A lot of that is because I feel anxious about something. And then I feel, like, mental pain and then I get very confused.

**Interviewer:** Okay. So tell me a little bit about the mental pain, what exactly is that.

**Participant:** Well, what I compare it to is, like, the mental equivalent of sitting on something you bruised or something. … It just feels like everything’s moving too fast; you overheat almost.

**Figure 4.**
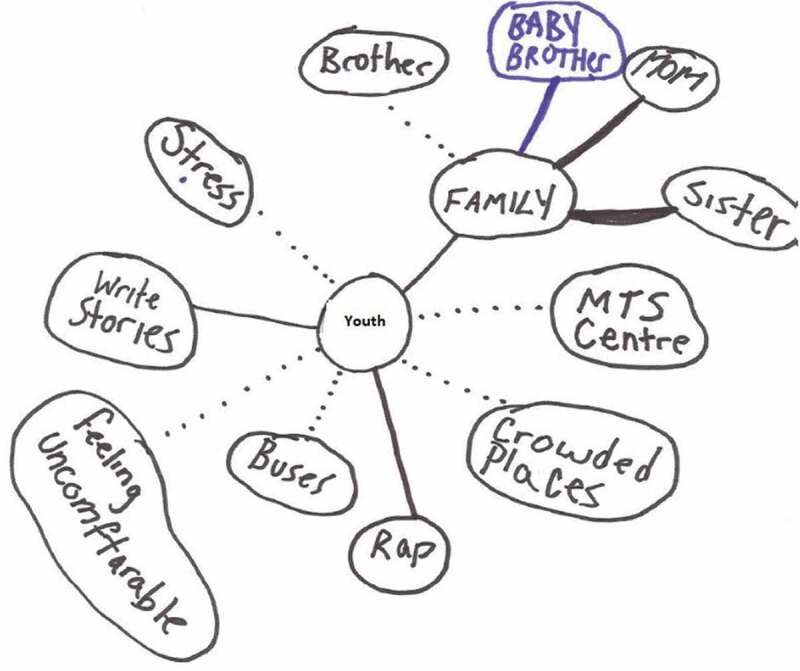
Feeling uncomfortable.

In addition to describing their experiences as *painful*, youth used terms such as *uncomfortable, unpleasant, heavy, hurt*, “*bad in my head*,” *suffering, broken*, and *empty* to describe their mental–emotional pain. They depicted their mental-emotional pain on their ecomaps, as revealed by one 15-year old (using a pseudonym) (Figure 4):

In describing her mental–emotional pain, another 19 year old explained as follows:

… it’s kind of like, like everything just shuts down but you just are trying to breathe but you can’t, and everything kind of seems just painful and just hard to do, and you just feel so scared. Um, like, I wish I could just explain it, like, when I just go full on like panic mode I just can’t breathe, I can’t talk, I just, and then I just cry because I can’t do anything and it’s just very scary, like I feel like with anxiety there’s panic attacks, and I had, like, a full-on blown panic attack at school one time and just, I just couldn’t do anything, I just sat there and I just couldn’t even pick up the phone and call my mom because everything just overwhelms your whole body, like the whole feeling.

**Figure 5.**
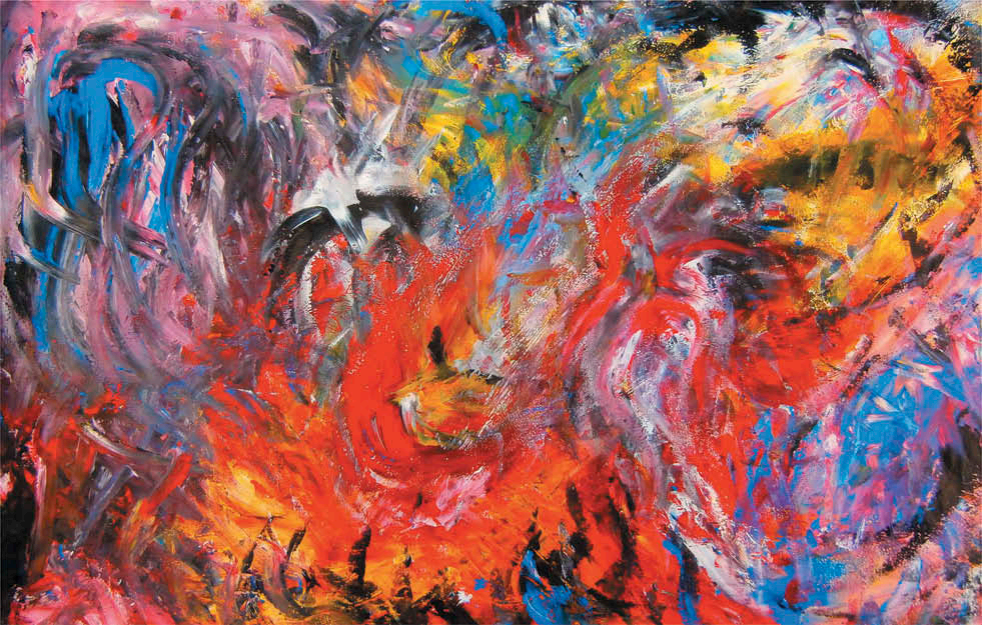
Demons.

Youth also relied on a number of metaphors to describe this mental–emotional pain. One youth prepared and then photographed her artwork to depict her experience of mental–emotional pain (Figure 5):

I called it “Demons.” … Um, I painted it when I wasn’t having a good time. And completely after I had no plans whatsoever and I just painted with my fingers and I just took colors and I swirled them around and mixed them on the paper. Um, and, uh, this is what came out of it, and I felt like it was really, it really showed what I was going through, and it really represented a lot of the things that were happening to me. Um, it’s chaotic in its nature like my mind was at that time … the connotation of, like, even without thinking about it the fire is coming up from the bottom. Leaching into everything.

Some young people in the study also shared their experiences with self-harm as a means of coping with the pain of living with an anxiety disorder. Overwhelmingly, those youth who engaged in self-harming behaviors, such as cutting, burning, scratching, or self-hitting, spoke of self-harm as a means of “transferring their pain.” Others felt that self-harm offered a break of sorts from the relentless nature of their emotional pain, as was the case with one 18-year-old participant, who shared,

Self-harm allows you to focus more on your physical pain than your mental pain.

Another young person, 14 years old, shared the following anecdote in her qualitative interview about using self-harm as a means of taking away one sort of pain, only to be left of a reminder of that pain:

**Interviewer:** And what, what made you cut, like, what was it about it?

**Participant:** I don’t know, just anything that, that would get to me, like if it was a big problem that got to me then I would cut. … Or like if people would yell at me, I’m not much of a yeller. … I don’t really like yelling at people. … And I guess when people yell at me that hits me, it’s like do you hate me or something, do you not like me, did I do something wrong? … And, I don’t know, it just scares me.

**Interviewer:** And so would cutting make it feel better?

**Participant:** I felt like the cutting took away the pain. … But then I regret it after I’m done it and I realize it does nothing, just left a scar to remind me or something. … Something that happened back then and, like, I tried to take it away but I couldn’t. It leaves a mark but it’s still there; it’s going to, like, haunt me.

#### Difficult interpersonal relationships: Social pain

**Figure 6.**
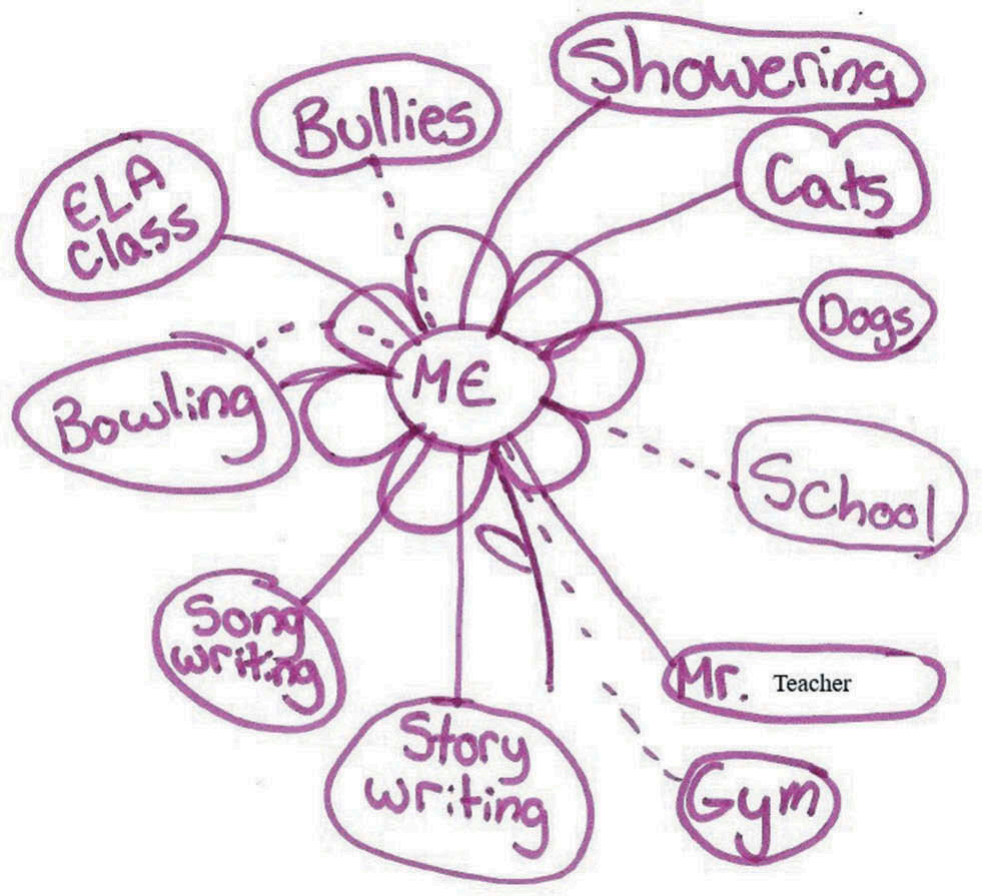
Social pain and bullying.

For many youth in the research study, interpersonal relationships were more difficult as a result of their anxiety. Many youth spoke of their experiences of bullying, of loss, and of rejection, and youth shared how painful it was to interact with others given this context. Though similar in some ways to mental–emotional pain, social pain by definition resulted from a lack of relationships with others or from interactions that were overwhelmingly negative in nature. As with physical pain, this social pain reduced their ability to participate in everyday life, with impacts at home, at school, and in their extracurricular activities. One 16-year-old drew an ecomap that depicted no friends and limited social supports but with an emphasis on the experience of bullying in her life (with a dotted line to represent the social pain) (Figure 6):

Similar to physical pain, with social pain came exhaustion. One youth, who was 17 years old at the time of the interview, talked about her social pain as follows:

I find it draining to talk to people, be around them and everything, it’s just, like, difficult and, like, like, I’ll seem very energetic around people and then people leave and I’m just, like, dead.

**Figure 7.**
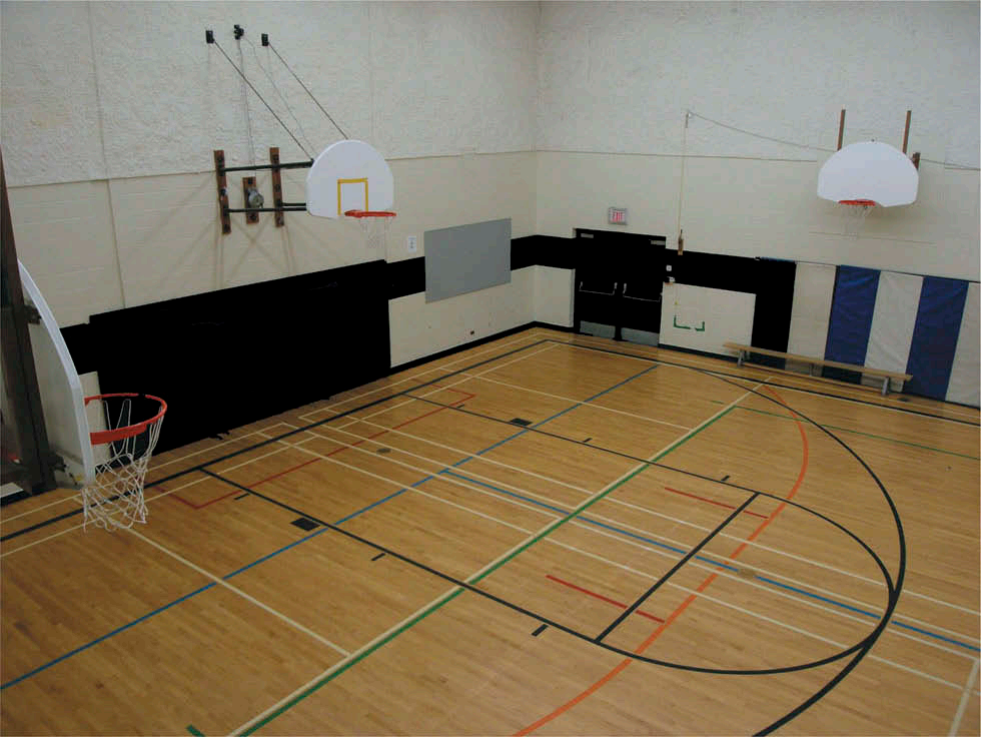
Painful memories.

Using photovoice, one 16-year-old participant returned to her elementary school to photograph the gymnasium, which represented her experiences of socially painful interactions, specifically bullying. In her words, she shared that the site was where “everything went to shit.” These experiences of bullying, which began in the gymnasium, were symbolic of her experience of anxiety. In hindsight, the youth recalled that as a result of these experiences, she missed months of school and that having to return to school after this absence was linked to her first experiences with self-harm (Figure 7).

This same participant, as with others, also discussed the social pain that resulted from having others, including those close to them, see them at such difficult periods in their lives. Similarly, another youth who dropped out of school as a result of her challenges living with mental illness shared,

I just feel constant pain from it [quitting school], like, I feel so sad about it, like, I regret it, like, it haunts me, like, I, I had something to do and it was my responsibility.

Mental health stigma was also an important contributing factor to the social pain experienced by youth participants in the project, including internalized stigma. Even the anticipation of stigma influenced youths’ participation in their worlds, and at times youth shared that they were afraid to voice certain experiences, including experiences of pain, for fear of how they would be perceived. However, youth also commented that by emphasizing the pain that comes with mental health, perhaps mental health stigma could be addressed.

#### Articulating their pain

For many young people in the study, despite articulating a subjective experience of pain that was complex and holistic, they often relied on physical descriptions of pain in order to create some sense of shared meaning with others. Their disclosure of experiences of pain and discomfort resulting from anxiety, at least initially, often was reduced to “I feel sick.” Repeatedly, parent participants in the study stated that they struggled to discern to what extent physical symptoms such as headaches or stomachaches were related to their mental health or to what extent they stood alone, demonstrating the hierarchy that often exists in experiences of pain. Such an approach was often met with frustration from youth, at not being believed or having their illness experience validated. In other cases, youth spoke of having to portray their pain in specific ways depending on the audience, in order to have their pain acknowledged or believed. One 13-year-old participant spoke adamantly about their desire to have health care providers that would listen to their experiences:

I need [a doctor] who will listen and so it’s not just in my head, well it is, but just not. … It’s not me making up all my pain.

Youth participants in the project discussed the difficulty that comes with articulating pain, particularly that which is invisible (social, mental–emotional), and communicating with others about their experiences living with invisible illnesses, such as anxiety disorders, depression, and other mental health conditions. A 22-year-old shared her difficulties in describing the impacts of anxiety on her life:

Like, I want to describe anxiety, like, this is what I’m going through, and it sucks. … ’Cause I think I can do more if anxiety wasn’t there, … like, it’s almost like a roadblock and that there’s so much physical pain that I feel that’s hard to describe at times, except for someone who’s actually living with it.

**Figure 8.**
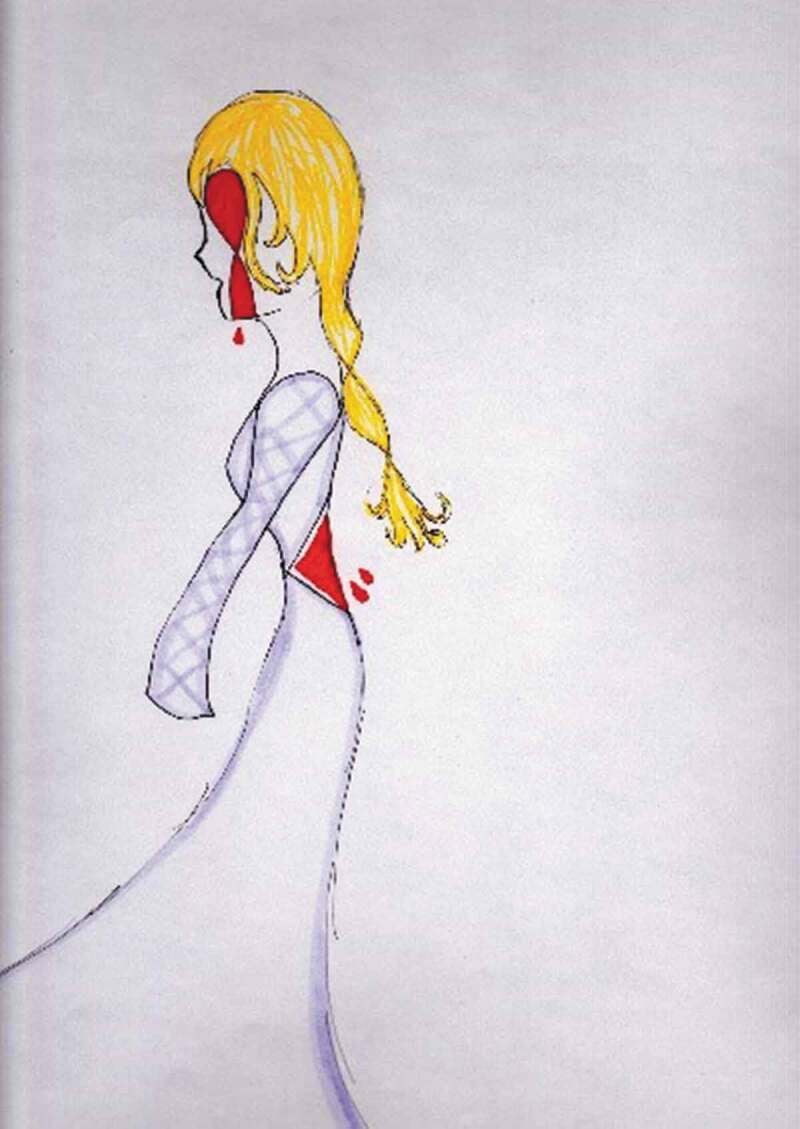
Using colors to articulate pain.

Another youth participant, 17 years old at the time of the interview, shared a photograph of her artwork as she recalled the difficulties that exist in articulating her pain and the ways in which art—from the drawings she creates to her choice of colors (especially the color red)—can serve as a tool for creating dialogue, helping her to communicate to others about how she feels (Figure 8).

Youth explicitly stated, and parents echoed, that it was painful for them to seek professional help only to be placed on waiting lists or not having those supports and services available to them, emphasizing the importance of timeliness in mental health supports and services.

**Figure 9.**
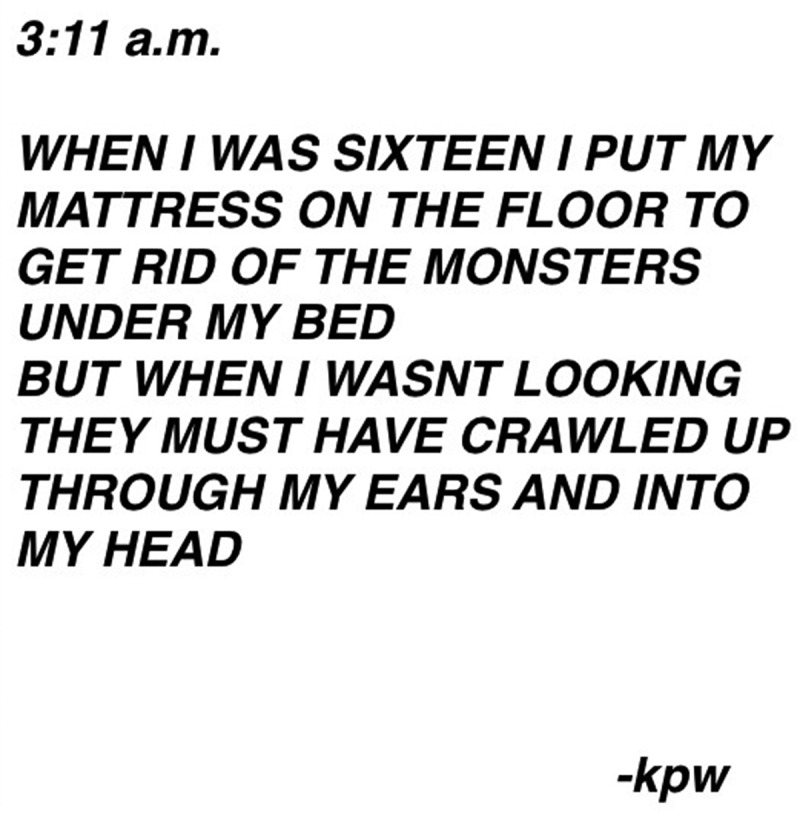
It still hurts.

Youth shared that attempts to manage their anxiety disorder meant addressing the various types of pain that were part of their experience. Many of the participants in the study were not necessarily looking for a *cure* to their anxiety or a pain-free existence; rather, there was an acceptance that some level of anxiety, and therefore pain, was going to be part of their lived experience. Instead, they emphasized coping strategies and management of their anxiety. One 19-year-old participant shared a screenshot from the Internet and summed it up as (Figure 9):

I think it [anxiety] just keeps going and, like, the pain, like, when you get a scratch or you hit your head or something, it still hurts, but you can’t feel it because you’ve gotten used to it, so. … Um, that happens all the time, like, I’ll finally get used to it [anxiety] and then it will just get worse and then. …

## Discussion

This study is one of the first to focus on the role of pain in the lived experiences of youth with anxiety disorders. Though some attention has been paid to the experiences of anxiety in youth with chronic pain,^6,8,68^ with anxiety viewed as secondary to the chronic pain experience, pain and anxiety have typically been treated as separate and distinct phenomena. By examining the role of pain in the lived experiences of youth with anxiety disorders, this study explores the ways in which anxiety and pain are interrelated. In youth experiencing pain, anxiety-related mental states (e.g., fear of pain and catastrophizing)^69^ and anxiety-provoking social contexts (e.g., isolation, interpersonal conflict)^21^ augment suffering. Yet youths’ narratives reinforced the multidimensional and interrelated nature of pain; for these youth, pain is experienced as much more than physical, also encompassing mental, emotional, and social dimensions. In some cases, mental and emotional pain lead directly to physical pain, with some youth engaging in self-harming behaviors as a means of coping with their mental and emotional pain. This multidimensionality expressed by youth is in stark contrast with the physical/psychological binary that is foundational to the biomedical model of pain.

Youth communicated their frustration with the seemingly artificial divisions of types of pain imposed by others, as well as the emphasis by both parents and the medical community on the physical aspects of pain. Although youth were frustrated by others’ focus on physical pain, they often resorted to rooting their descriptions of their pain experiences in physical terms in order to make their voices heard and to have their experiences acknowledged and believed. This strategy reflects the hierarchical nature of different types of pain in the medical community, with physical pain receiving much more attention and care than pain that is mental, emotional, or social in nature. Even though the International Association for the Study of Pain’s proposed new definition of *pain* describes it as an aversive sensory and emotional experience,^2^ the mental and emotional aspects of pain are rarely considered in pediatric pain research and practice. The mental health of children and youth is often referred to as the “orphans’ orphan” of health care, signifying the lack of attention and understanding directed to pain that is not physical in nature.^17^ This focus on physical pain results in under- and unaddressed emotional, mental, and social pain experiences of youth with anxiety disorders. Challenging this hierarchy is crucial in order to provide services that address all dimensions of youth pain, which is necessary in order to promote youth well-being.

Similar to the narratives of young people living with chronic pain, youth with anxiety described their pain as often invisible in nature, indeterminate, unpredictable, and complex in its course.^1,11,70^ The invisible nature of the pain experienced by youth with anxiety disorders is mirrored in the unseen work that youth engage in in order to cope and manage with both their anxiety and their associated pain.

This study’s findings support the use of the biopsychosocial model of pain in both research and clinical practice. Considered a patient-centered approach, the biopsychosocial model considers how all of the aspects of one’s life converge to influence the development, experience, and maintenance of pain.^12,40^ This approach highlights the need for clinicians to recognize and address all types of pain—physical, emotional, mental, and social—that may manifest in a young person’s life. Ways in which clinicians can begin to address the multidimensionality of pain include standardized pain assessment tools and allowing young people to share their experiences without limiting attention to physical pain experiences.

Prior research in the areas of both youth anxiety disorders and youth pain have been primarily quantitative in nature, limiting our understanding of the complex reality of the lived experiences of youth living with these conditions and the ways in which anxiety and pain may both play a role in youths’ experiences. As previously noted, the interview guides used in this study did not focus on youths’ pain experiences. Rather, the qualitative, arts-based nature of this study allowed youth to reflect and share their thoughts on their lived experiences with anxiety, allowing the significance of pain in these lived experiences to come through. As demonstrated by their use in this study, arts-based methods allow youth greater control over what is discussed in the research setting, affording youth the opportunity to drive the conversation and call attention to aspects of their experiences that researchers may overlook.^71,72^ In this way, qualitative, arts-based methods in research with youth can elicit both more and different information than standard interview or survey techniques.^73,74^ Additionally, in previous work, youth have noted the ways in which arts-based methods have facilitated the communication process^31^; as Thomson noted, “Images communicate in different ways than words.”^74(p11)^ In the health care research context, qualitative, arts-based methods can help youth find external expression for the subjective experience of pain by acting as a conduit between language and affective state.^1,75^ Arts-based representations of health knowledge are also useful for thinking across epistemologies,^76^ such as with the acute pain model and/or the biomedical perspective.

### Limitations and future research

This study is one of few to explore the pain experiences of youth with anxiety disorders. Though the goal of purposive sampling is to maximize various or diversity,^52,53^ participants in this study were predominantly white and female, limiting transferability of findings to other populations. Future work should include more diverse groups of youth in order to broaden our understanding of pain in the experiences of youth with anxiety disorders.

Additionally, more work is needed with youth with a variety of mental disorders in order to continue to broaden our understanding of the experience of pain in those with mental illness, including its multidimensional and interrelated nature. Further insight into the complex, lived experiences of youth with mental disorders will provide support for a comprehensive, evidence-based approach to pain assessment and management in pediatric patients experiencing pain.

## Conclusions

By utilizing qualitative, arts-based methods, a multidimensional picture of pain in young people with anxiety was revealed. Anxiety in youth needs to be approached from a biopsychosocial perspective in order to capture the holistic experience. Qualitative and arts-based methods can serve as “catalysts for change,”^73(p48)^ highlighting aspects of youths’ lived experiences that reveal structural and systemic barriers to their health and well-being,^31,77^ prompting advocacy and action. This work reinforces the need for the use of qualitative and arts-based approaches to understanding pain experiences in young people.
